# Association of Proteinuria With Disease Stage and Treatment Response in Patients With Lymphoma: A Prospective Cohort Study

**DOI:** 10.7759/cureus.105686

**Published:** 2026-03-23

**Authors:** Nitin Kumar, Sudhir K Atri, Vikas Chaudhary, Piyush Malik, Kuhu Chatterjee, Gaurav Arora, Monika Kumari

**Affiliations:** 1 Department of Medicine, Pandit Bhagwat Dayal Sharma Post Graduate Institute of Medical Sciences, Rohtak, IND; 2 Department of Microbiology, Institute of Medical Sciences, Banaras Hindu University, Varanasi, IND

**Keywords:** chemotherapy, hodgkin lymphoma, non-hodgkin lymphoma, proteinuria, urinary protein

## Abstract

Background

Lymphomas are hematologic malignancies that may involve the kidneys, with proteinuria representing a potential manifestation of renal involvement or paraneoplastic processes. The relationship between proteinuria, disease stage, and treatment response in lymphoma remains incompletely understood.

Objectives

The primary objective was to assess the association between proteinuria and disease stage in patients with lymphoma. Secondary objectives included evaluating changes in proteinuria following completion of standard chemotherapy or induction remission, whichever occurred earlier.

Methods

This prospective cohort study included 34 adult patients with biopsy-confirmed lymphoma at a tertiary care center over 12 months. Staging was performed using PET-CT according to the Lugano classification. Twenty-four-hour urinary protein was measured at diagnosis and after completion of chemotherapy or induction remission, whichever occurred earlier. Proteinuria was defined as >150 mg/24 hours. Associations between proteinuria, disease stage, and treatment response were analyzed using appropriate statistical methods.

Results

Of the 31 patients included in the final analysis, proteinuria was observed in seven out of 31 (22.6%), including five out of 23 (21.7%) with non-Hodgkin lymphoma and two out of eight (25%) with Hodgkin lymphoma. All patients with proteinuria had advanced-stage disease, although this association was not statistically significant (p=0.161). Mean 24-hour urinary protein was higher in advanced-stage disease compared to early-stage disease (85 mg/day vs. 52 mg/day; p=0.069). Among patients with proteinuria, one out of seven (14.3%) died during treatment. Among the remaining patients, two out of six (33.3%) achieved a complete response, and four out of six (66.7%) had a partial response, whereas patients with normal protein levels demonstrated equal proportions of complete and partial response. These differences were not statistically significant (p=0.657). Paired analysis demonstrated a significant reduction in proteinuria following treatment (p=0.031).

Conclusion

Proteinuria decreased significantly following chemotherapy and may serve as a simple, non-invasive marker for monitoring treatment response in lymphoma patients. Although higher proteinuria was observed in advanced-stage disease and was associated with poorer treatment response, these findings were not statistically significant. Larger studies are required to establish the prognostic value of proteinuria in lymphoma.

## Introduction

Lymphomas are a category of blood cancers that originate from lymphoid cells. Survival data for lymphoma in the Indian population indicate that for those who complete treatment, outcomes for Hodgkin lymphoma (HL) are comparable to developed nations, while non-Hodgkin lymphoma (NHL) outcomes can be less favorable, partly due to advanced stages at diagnosis and treatment access issues [1,2]. B-cells, T cells, and infrequently natural killer cells can give rise to them. Although it can affect any area of the body, lymph nodes are the primary source of lymphoma. The cause of the rise in lymphoma cases during the last few years is uncertain. Although the exact etiology of lymphoma remains unclear, certain individuals are more susceptible to the condition, including those with HIV/AIDS and those who are infected with *Helicobacter pylori*, Epstein-Barr virus, or human T-lymphotropic virus [3]. The two primary types of lymphomas are NHLs and HLs. NHL is the most prevalent hematological malignancy in the world. NHL accounts for over three out of every four cancer diagnoses and fatalities. Lymphoma is classified according to the Lugano system (an updated version of the Ann Arbor classification) and is assessed using PET-CT. Diagnosis is based on histopathological evaluation, typically performed using an excisional lymph node biopsy [4,5]. While early-stage disease often has a favorable prognosis, advanced-stage, bulky, or high-grade cases require more intensive therapy and have different prognostic factors [6].

The incidence of both HL and NHL is rising across India and Asia, with NHL representing a significant and increasing proportion of hematological malignancies. The age-adjusted rates of incidence for NHL in men and women in India are 2.9 per 100,000 and 1.5 per 100,000, respectively. In comparison to developed countries, notable differences in presentation within India include a median age of 54 years (nearly a decade younger), a higher male-to-female ratio, a greater percentage of patients exhibiting B-symptoms, a poorer Eastern Cooperative Oncology Group (ECOG) performance status (≥2) at the time of diagnosis, a higher prevalence of diffuse large B-cell lymphomas, a lower occurrence of follicular NHL, and T-cell type lymphomas [2]. HL in India, similar to other Asian populations, is marked by a considerable prevalence among children and young adults, commonly showing the mixed cellularity histological subtype, unlike the predominance of nodular sclerosis found in Western countries. India reports more than 11,000 new cases each year, with a five-year survival rate of around 83-84% [7]. The symptoms of lymphoma differ from patient to patient based on which organs are involved, the rate of tumor progression, and the location of the lymphoma cells. Individuals often show swollen lymph nodes in areas such as the neck, groin, or armpits. Those with intermediate or high-grade lymphoma might also suffer from additional systemic symptoms, which can include severe fevers, night sweats, unintended weight loss, and a general sense of fatigue [3]. Lymphoma can affect various organs, including the kidneys. Kidney issues, particularly renal infiltration due to lymphoma, are frequently found during autopsies (30-60% of cases), but are recognized in only 3-8% of individuals while alive. Chronic kidney disease (CKD) affects around 34.5% of patients with lymphoma [8]. The typical causes include prerenal azotemia (due to dehydration or a lack of fluid volume), acute kidney injury (AKI), and very rarely, primary renal lymphoma [9].

Proteinuria may precede the diagnosis of lymphoma or may occur simultaneously. Paraneoplastic glomerulopathy has been reported in patients with malignancy [10]. Persistent proteinuria can be a paraneoplastic syndrome, and it is important to consider HL in the diagnosis, since this is essential for the management of both conditions; there is evidence that proteinuria tends to resolve following treatment in patients with lymphoma [11]. Since proteinuria appears to be the first sign of renal involvement in lymphoma patients and disappears with effective lymphoma treatment, only a few studies have observed a relationship between the extent of proteinuria and stages of lymphoma [12]. We undertook this study to find out the correlation (if any is present) between proteinuria in lymphoma patients and various stages of lymphoma.

## Materials and methods

This was a single-center, longitudinal, prospective cohort study conducted at a tertiary care teaching hospital in northern India. The institution has a 10-bed hematology unit, with patients also managed in shared medical wards under specialist supervision. A total of 34 patients were enrolled over a 12-month period from December 2022 to December 2023. All patients had a confirmed lymphoma diagnosis (biopsy and immunohistochemistry (IHC) proven), including all forms of HL and NHL. The inclusion criteria included patients over 18 years of age, newly diagnosed lymphomas confirmed by lymph node biopsy, bone marrow biopsy, and IHC, and informed consent from the patient. Patients with pre-existing diabetes or hypertension, or CKD, patients with pre-existing advanced co-morbid conditions where the standard chemotherapy protocol was not applicable, were excluded from the study. Patients with malignancies other than lymphoma were also excluded.

A detailed history, complete physical examination, and appropriate investigations were done for all patients. Diagnosis was confirmed by histopathological examination of lymph node biopsy/trucut biopsy and IHC markers, followed by a battery of tests like PET scan to characterize the type and stage of lymphoma. Staging was done on the basis of PET-CT findings according to the Lugano classification [5]. Standard instructions were provided to ensure proper 24-hour urine collection. The amount of protein excreted in urine over 24 hours was measured quantitatively by the colorimetric method using pyrogallol red on diagnosis and after six months of standard chemotherapy or completion of induction chemotherapy, whichever was earlier. Twenty-four-hour urine protein levels >150mg/24 hours were considered abnormal [13]. After final staging, induction chemotherapy was started. Standard treatment was given according to the subtypes of the lymphoma: HL - adriamycin, bleomycin, vinblastine, and dacarbazine (ABVD); NHL (B-cell) - rituximab, cyclophosphamide, doxorubicin, vincristine, and prednisolone (RCHOP) or bendamustine and rituximab (BR); T cell - etoposide, cyclophosphamide, doxorubicin, vincristine, and prednisolone (ECHOP) [14]. Response assessment was done as per Lugano criteria for metabolic response assessment on fluorodeoxyglucose (FDG)-PET-CT [5]. Complete response (CR) was defined as complete metabolic response on PET-CT (Deauville score 1-3), with resolution of all metabolically active disease and no evidence of residual disease. Partial response (PR) was defined as a reduction in metabolic activity of previously involved sites (Deauville score 4-5 with reduced uptake compared to baseline) and a ≥50% decrease in the sum of the products of the diameters of measurable lesions.

Primary outcomes were assessed by correlation between urine protein excretion at 24 hours and the lymphoma stage, as well as the effect of lymphoma treatment on urine protein excretion at 24 hours. The secondary outcome was determined by an association between the different phases of lymphoma and remission.

Statistical analysis

Data were collected and analyzed using Microsoft Excel (Microsoft Corp., Redmond, WA, USA). Descriptive statistics were used to summarize baseline characteristics, with continuous variables expressed as mean±standard deviation and categorical variables presented as frequencies and percentages. Normality of continuous variables was assessed using visual inspection and appropriate statistical methods, and suitable parametric or non-parametric tests were applied accordingly. Associations between categorical variables were evaluated using the chi-square test or Fisher’s exact test where appropriate. Comparisons between independent groups were performed using the Mann-Whitney U test. Paired comparisons of 24-hour urinary protein levels before and after chemotherapy were assessed using the Wilcoxon signed-rank test. Patients who died during the study period were excluded from the final analysis of treatment response but were included in baseline and descriptive analyses. Missing data were handled using complete case analysis. Odds ratios (ORs) with 95% confidence intervals (CIs) were calculated where applicable. A p-value<0.05 was considered statistically significant.

Ethical clearance

The study was duly approved by the ethics committee of the Institute (Biomedical Research Ethics Committee, Pt. B. D. Sharma PGIMS/UHS, Rohtak, with approval BREC/22/TH/Med.-02, dated 15/11/2022). All methods conducted in the study that involved human subjects adhered to the ethical principles established by the institutional and/or national research committee, as well as the 1964 Helsinki Declaration and its later amendments or comparable ethical guidelines.

## Results

The baseline characteristics of the study population are summarized in Table 1. The mean age was 49.9±17 years, with a male predominance (24/34, 70.6%). The mean height, weight, and body mass index were 164.36±6.75 cm, 57.94±10.05 kg, and 21.35±2.53 kg/m^2^, respectively. The mean body surface area was 1.86±0.19 m^2^. Lymphadenopathy was the most common clinical finding (31/34, 91.2%), followed by fever (22/34, 64.7%) and pallor (8/34, 23.5%). Most patients presented with advanced disease, with stage IVB being the most frequent (15/34, 44.1%), followed by stage IIIB and IVA (6/34, 17.6% each). During the study period, four out of 34 patients (11.8%) died, of whom three died before completion of evaluation. Among the remaining 31 patients included in the final analysis, 23/31 (74.2%) had NHL, and eight out of 31 (25.8%) had HL.

**Table 1:** Baseline demographic and clinical characteristics of the study population (N=34). Data are presented as n (%) unless otherwise stated. Age is expressed as mean±SD. Proteinuria (>150 mg/24 h) was assessed by 24-hour urine collection and was available for 31 patients, as three patients expired before evaluation.

Parameter	Number (N=34)	Percentage (%)
Gender		
Male	24	70.6
Female	10	29.4
Age years (Mean±SD)	49.9±17	-
<18 years	1	2.9
18-30 years	4	11.7
30-45 years	7	20.6
45-60 years	12	35.3
>60 years	10	29.4
Presenting clinical features		
Lymphadenopathy	31	91.2
Fever	22	64.7
Pallor	8	23.5
Staging		
IA	2	5.9
IB	1	2.9
IIA	4	11.8
IIB	0	0
IIIA	0	0
IIIB	6	17.6
IVA	6	17.6
IVB	15	44.1
Proteinuria (mg/24 hours)		
>150 mg	7	22.6
<150 mg	24	77.4
Response to treatment		
Complete response (CR)	14	41.2
Partial response (PR)	16	47
Expired	4	11.8

**Figure 1:**
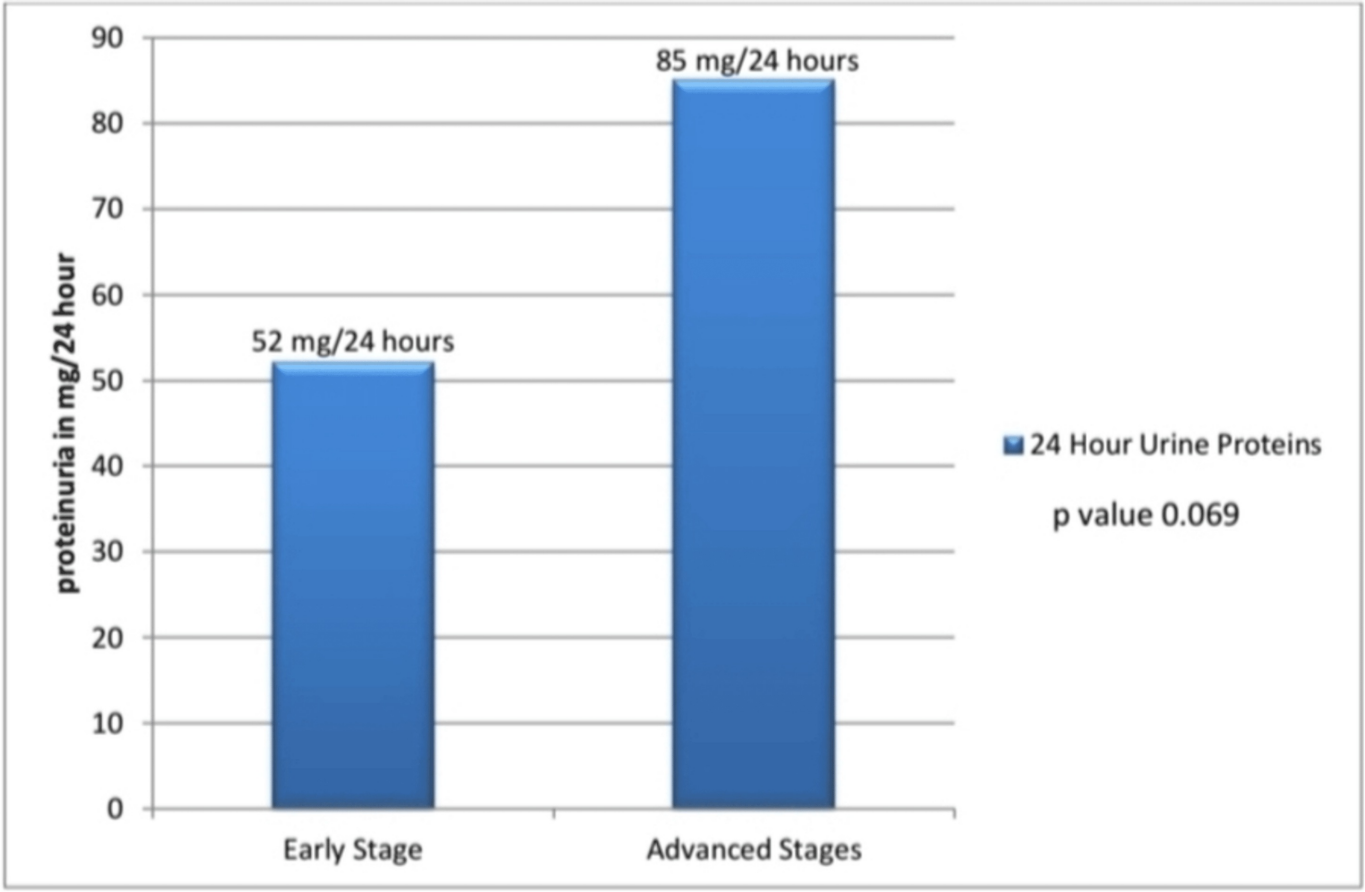
Comparison of the mean 24-hour urine protein in the early and advanced stage groups. Early-stage group refers to stages IA, IB, and IIA. The advanced-stage group refers to stages IIB, IIIA, IVA, and IVB.

Proteinuria was observed in seven out of 31 patients (22.6%), including five out of 23 (21.7%) with NHL and two out of eight (25%) with HL. All affected patients had advanced-stage disease (IIB-IVB), although this association was not statistically significant (p=0.161), as shown in Table 2. Mean 24-hour urinary protein was higher in advanced-stage disease (85 mg/day) than in early-stage disease (52 mg/day), showing a non-significant trend (p=0.069), as illustrated in Figure 1.

**Table 2:** Association between stages, proteinuria, and treatment response. Values are presented as n (%). Associations were analyzed using Fisher's exact test. Odds ratios (ORs) with 95% confidence intervals (CIs) are reported. The wide CIs reflect the small sample size and the presence of zero counts in one comparison group, resulting in imprecise interpretations; * p<0.05 was considered significant.

Parameter	24-hour proteinuria		p-value*	OR (95% CI)
	Normal, n (%)	Elevated, n (%)		
Stage				
Early	7 (29.2)	0 (0)	0.161	5.76 (0.29-115.91)
Advanced	17 (70.8)	7 (100)		
Response				
Complete	12 (50.0)	2 (33.3)	0.657	2.00 (0.31-13.06)
Partial	12 (50.0)	4 (66.7)		

**Figure 2:**
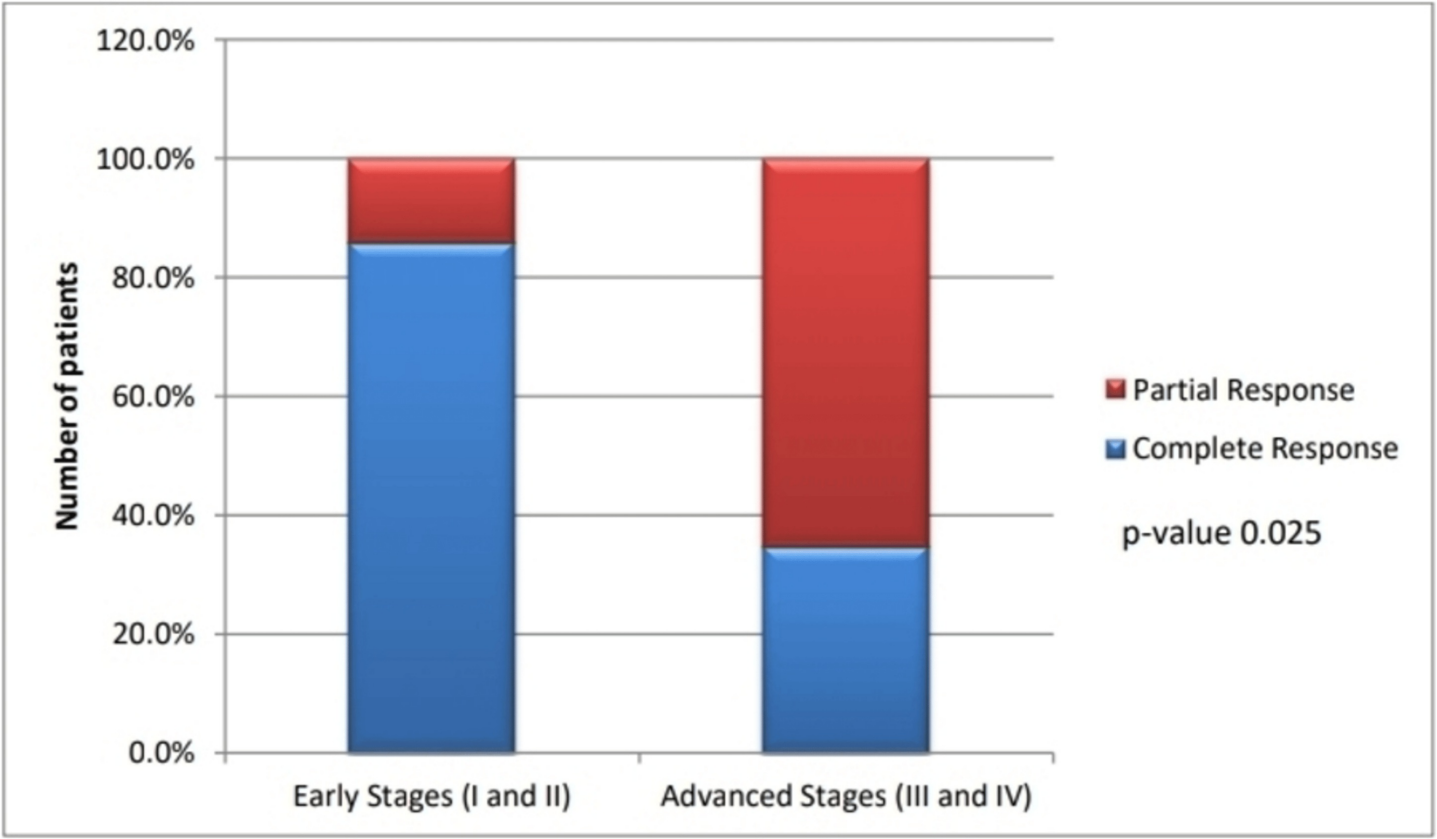
Association between staging and treatment response. The figure highlights the significantly higher complete response observed among early-stage lymphoma than among advanced-stage lymphoma.

Among all patients (n=34), 14/34 (41.2%) achieved CR, 16/34 (47%) had PR, and four out of 34 (11.8%) died during treatment. CR was significantly more frequent in early-stage disease (IA-IIA) compared to advanced-stage disease (IIB-IVB) (85.7% vs. 34.7%), while PR was more common in advanced-stage disease (65.3% vs. 14.3%) (p=0.025), as illustrated in Figure 2.

Among patients with elevated proteinuria (n=7), one patient died before completion of chemotherapy. Among the remaining six patients, two out of six (33.3%) achieved CR, while four out of six (66.7%) had PR. All patients with proteinuria had advanced-stage disease (7/7, 100%), whereas none of the early-stage patients had elevated proteinuria; however, this association was not statistically significant (p=0.161; OR 5.76, 95% CI 0.29-115.91). Patients with normal 24-hour urinary protein levels demonstrated equal proportions of CR and PR (50% each), and the difference in treatment response between groups was not statistically significant (p=0.657; OR 2.00, 95% CI 0.31-13.06), as shown in Table 2.

**Figure 3:**
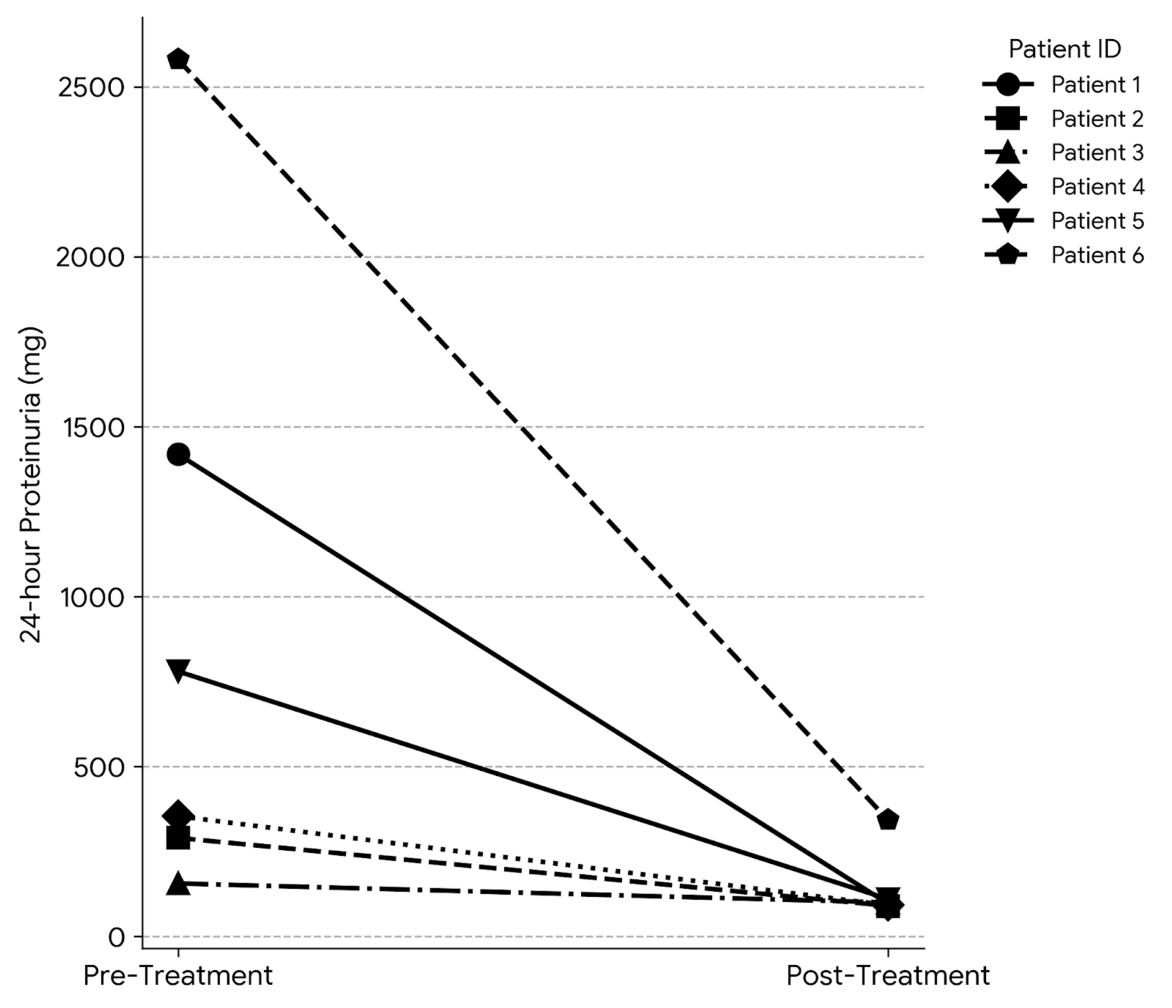
Change in 24-hour urinary protein levels from diagnosis to post-chemotherapy evaluation in patients with elevated baseline proteinuria (n=6). Paired measurements of 24-hour urinary protein excretion for six patients with elevated baseline proteinuria are shown at diagnosis and after completion of chemotherapy. Each line represents an individual patient, demonstrating a decline in proteinuria following treatment. One patient with elevated proteinuria expired before follow-up assessment.

Paired measurements of 24-hour urinary protein were available for six patients with elevated baseline proteinuria and are represented in Figure 3. Following chemotherapy, mean proteinuria decreased from 929.8 mg/day at diagnosis (range: 156-2580 mg/day) to 138.3 mg/day after treatment (range: 90-342 mg/day), corresponding to a mean reduction of 791.5 mg/day (95% CI: approximately 100-1480 mg/day). The decrease was statistically significant on paired analysis (Wilcoxon signed-rank test, p=0.031), although interpretation should be made cautiously given the small sample size.

## Discussion

In the present study, proteinuria was observed in seven out of 31 patients (22.6%), including five out of 23 (21.7%) with NHL and two out of eight (25%) with HL. In similar studies from the literature, the prevalence of proteinuria was reported to be 15.2% in NHL patients [15]. Ubukata M et al. reported 34.5% prevalence of CKD and 14.5% prevalence of proteinuria in lymphoma patients [8]. A similar study from France by Kohn M et al. reported that kidney failure, indicated by a glomerular filtration rate of less than 60 mL/min/1.73 m^2^, was observed in 47% of the patients [16]. In the present study, a greater proportion of patients with advanced-stage disease (IIB-IVB) had elevated 24-hour urinary protein levels compared to those with early-stage disease (IA-IIA), although the difference was not statistically significant (p=0.161). Mean 24-hour urinary protein levels were also higher in advanced-stage disease, showing a non-significant trend (p=0.069). These findings are consistent with previous reports, including the study by Dilek et al., which demonstrated higher urinary albumin excretion (UAE) in patients (31.2 µg/min) compared to controls (5.6 µg/min) [17]. Similar studies from the literature have also shown that patients in the advanced stages tended to have a higher mean UAE than patients in the early stages [17,18]. This suggests that 24-hour urinary protein levels can be correlated with the stages of lymphoma; however, strong evidence is lacking and requires more extensive research.

Patients having normal proteinuria responded better to therapy as compared to patients having elevated urinary protein; however, results were not statistically significant (p=0.657). Among patients with elevated proteinuria, a higher proportion had a PR, and one patient died during treatment, whereas no deaths occurred in the normal proteinuria group. This suggests a possible association between proteinuria and poorer treatment response and mortality; however, the findings were not statistically significant. Similar other studies from the literature in patients with lymphomas have also shown that patients having proteinuria had a higher risk for mortality as compared to the normal patients [15,19,20]. In the present study, proteinuria decreased in all patients after treatment. Among the six patients with paired measurements, the mean 24-hour urinary protein at diagnosis was 929.8 mg/day, which significantly decreased to 138.3 mg/day after chemotherapy (p=0.031). Similar studies from the literature have also shown improved outcomes and decreased proteinuria in lymphoma patients after treatment [8,19]. The study done by Pedersen LM et al. reported that the median value of UAE after treatment was considerably reduced compared to the median value before treatment (23.0 vs. 38.0 mg/min; p< 0.0001) [12]. This suggests that proteinuria may serve as a simple and affordable laboratory marker to monitor treatment response in lymphoma patients. These findings should be interpreted as exploratory and hypothesis-generating, given the limited sample size and potential confounding factors.

Study limitations

This study has several limitations. HL and NHL were analyzed collectively despite known differences in biological behavior, prognostic implications, and treatment response, and the heterogeneity within NHL subtypes may have introduced confounding. The relatively small sample size (n=34), particularly in the HL subgroup, precluded subgroup analyses and limited generalizability. Additionally, the single-center design and inclusion of predominantly referred patients may introduce selection bias and reduce representation of the broader population. Proteinuria, as a marker of renal involvement, may be influenced by multiple factors, and complete exclusion of potential confounders was not feasible; moreover, multivariable adjustment was not performed. The absence of multivariable analysis limits the ability to determine whether proteinuria is independently associated with disease stage or treatment response. The potential nephrotoxic effects of anthracycline-based chemotherapy, particularly doxorubicin, were not accounted for, and renal function parameters such as serum creatinine and estimated glomerular filtration rate were not systematically monitored. Therefore, treatment-related proteinuria cannot be entirely excluded, although this effect is likely non-differential given similar treatment regimens across patients.

Larger, multicentric studies with adequate sample size and subtype-specific analyses are required to validate these findings and better define the prognostic significance of proteinuria in lymphoma.

## Conclusions

Treatment resulted in a significant reduction in proteinuria among lymphoma patients with elevated baseline 24-hour urinary protein excretion. Although higher proteinuria was observed in advanced-stage disease and showed a trend toward poorer treatment response, these associations were not statistically significant, and no significant correlation with disease stage was found. Overall, proteinuria may serve as a simple, non-invasive marker for monitoring treatment response in lymphoma; however, its prognostic utility remains uncertain and requires validation in larger, well-designed studies.
